# MYC inhibition reprograms tumor immune microenvironment by recruiting T lymphocytes and activating the CD40/CD40L system in osteosarcoma

**DOI:** 10.1038/s41420-022-00923-8

**Published:** 2022-03-15

**Authors:** Kuo Jiang, Qianfeng Zhang, Yong Fan, Jia Li, Jitao Zhang, Wentao Wang, Jinzhu Fan, Yunshan Guo, Shichang Liu, Dingjun Hao, Yongxiang Wang, Lei Wang, Lequn Shan

**Affiliations:** 1 Department of Spine Surgery, Honghui Hospital, Xi’an Jiaotong University, Xi’an, China; 2 Department of Obstetrics and Gynecology, Xijing Hospital, Fourth Military Medical University, Xi’an, China; 3 Department of Bone Microsurgery, Honghui Hospital, Xi’an Jiaotong University, Xi’an, China; 4 Department of Orthopedics, Northern Jiangsu People’s Hospital, Affiliated Hospital of Nanjing University Medical School, Yangzhou, China; 5 Clinical Medical College, Yangzhou University, Yangzhou, China; 6 Department of Medical Research Center, Northern Jiangsu People’s Hospital, Affiliated Hospital of Nanjing University Medical School, Yangzhou, China

**Keywords:** Immune evasion, Bone cancer

## Abstract

The efficacy of immune checkpoint blockade (ICB) therapy depends on sufficient infiltration and activation of primed tumor-specific cytotoxic T lymphocytes (CTLs) in the tumor microenvironment. However, many tumor types, including osteosarcoma, mainly display immune-desert or immune-excluded phenotypes, which are characterized by a lack of tumor-infiltrating lymphocytes and a poor response to ICB monotherapy. Thus, novel therapeutic strategies are urgently needed to surmount these obstacles. In this study, we found that the expression of the c-Myc oncogene is negatively correlated with the T cell infiltration rate in osteosarcoma. Pharmacological inhibition of c-Myc with JQ-1 significantly reduced tumor burden and improved overall survival in an immunocompetent syngeneic murine model of osteosarcoma (K7M2). A mechanistic study revealed that JQ-1 administration dramatically reprogrammed the tumor immune microenvironment (TIME) within K7M2 tumors. On the one hand, JQ-1 can promote T cell trafficking into tumors by increasing the expression and secretion of T cell-recruiting chemokines. On the other hand, JQ-1 is capable of facilitating crosstalk between antigen-presenting dendritic cells and T cells through the CD40/CD40L costimulatory pathway, leading to activation of tumor-specific CTLs. Combined treatment with anti-PD-1 antibody and JQ-1 resulted in more pronounced tumor regression than either monotherapy, showing an obvious synergistic effect. These findings uncover for the first time that c-Myc inhibition can promote T cell infiltration and activation in osteosarcoma in multiple ways, delivering a one-two punch for modulating TIME. The present work also provides the basis for establishing c-Myc inhibitor and ICB coadministration as a novel therapeutic regimen for patients with osteosarcoma.

## Introduction

Osteosarcoma is the most common primary malignant bone tumor that occurs in children and adolescents and is characterized by rapid growth, local aggressiveness, and early metastasis. Although the 5-year survival rate of patients with localized osteosarcoma was improved to 65–70% by standard multiagent chemotherapy combined with surgical resection, the overall survival rate in osteosarcoma has remained virtually unchanged over the past 30 years [1]. Current therapies have limited effects on the treatment of metastatic or recurrent osteosarcoma, resulting in a poor prognosis with a survival rate of <20% [2]. Moreover, the quality of life and mental health of osteosarcoma patients who undergo amputation are seriously impaired. Therefore, novel therapeutic strategies are urgently needed to improve the clinical outcome in osteosarcoma.

The immune system is capable of recognizing and eliminating newly arising tumor cells but is held in check by inhibitory receptors expressed on immune cells. These immune checkpoint pathways, which are crucial for maintaining self-tolerance and limiting collateral tissue damage during antimicrobial immune responses, can be exploited by cancer cells to evade immune destruction [3]. In the past decade, immune checkpoint blockade (ICB) therapy has attracted a great deal of attention and become a major focus in current cancer research [4]. Monoclonal antibodies (mAbs) targeting cytotoxic T lymphocyte-associated antigen 4 (CTLA-4) or the programmed cell death protein 1 pathway (PD-1/PD-L1) are already being applied clinically to treat a variety of human cancers, especially advanced solid tumors [5, 6]. However, the utility of this approach for osteosarcoma has lagged far behind its utility in other cancers [7]. A phase 2 clinical trial in patients with advanced osteosarcoma showed limited activity of PD-1 inhibition and pointed out the necessity of combination therapy [8]. Typically, the efficacy of ICB therapy depends on sufficient infiltration and activation of primed tumor-specific CTLs in the tumor microenvironment [9]. However, many types of cancer, including osteosarcoma, mainly display immune-desert or immune-excluded phenotypes [10–12], which are characterized by a lack of TILs and a poor response to ICB monotherapy. Therefore, we speculated that if a drug could promote the cancer-immunity cycle by enhancing T cell infiltration and activation, it would greatly improve the response rate and clinical efficacy of ICB therapy.

The ubiquitous deregulation of the c-Myc (also called MYC) oncogene in human cancers makes it an intriguing therapeutic target [13]. Recent studies have demonstrated that c-Myc not only plays critical roles in cell growth, apoptosis, and cancer metabolism but also functions as a key regulator of the tumor microenvironment and immune response [14–16]. Among the many immune cell subsets regulated by c-Myc, T lymphocytes are of particular concern. Overexpression of c-Myc in stimulated T cells generated the canonical T cell immune-response pattern of rapid growth followed by loss of most cells [17], while c-Myc depletion induced by epigenetic therapy modulated the exhausted T cell phenotype toward memory and effector T cell phenotypes [18]. Moreover, small-molecule c-Myc inhibitors increased tumor immune cell infiltration, upregulated PD-L1 on tumor cells, and sensitized tumors to anti-PD-1 immunotherapy [19]. It is worth noting that the regulation of PD-L1 expression on the surface of T cells by c-Myc is tumor-type-dependent [14, 19, 20], suggesting that c-Myc-mediated immune regulation deserves more in-depth investigation in different cancers. In the field of osteosarcoma research, it was found that c-Myc was overexpressed in a high percentage of localized tumors and metastases and that higher c-Myc expression correlated significantly with recurrence and poor prognosis in patients with osteosarcoma [21, 22]. However, the relationship between tumor immune suppression and c-Myc overexpression in osteosarcoma remains largely unexplored, hindering the application of c-Myc inhibitors in combination immunotherapy for osteosarcoma.

Here, we report that pharmacological inhibition of c-Myc in osteosarcoma on the one hand promotes T cell trafficking into tumor beds by increasing the expression and secretion of T cell-recruiting chemokines and on the other hand facilitates crosstalk between antigen-presenting DCs and T cells through the CD40/CD40L costimulatory pathway. This study revealed for the first time that c-Myc inhibition can enhance T cell infiltration and activation in osteosarcoma in multiple ways, thus showing great potential for establishing c-Myc inhibitor and ICB coadministration as a novel therapeutic regimen for patients with osteosarcoma.

## Results

### c-Myc is highly expressed in human osteosarcoma and negatively correlated with the T cell infiltration rate

**Fig. 1:**
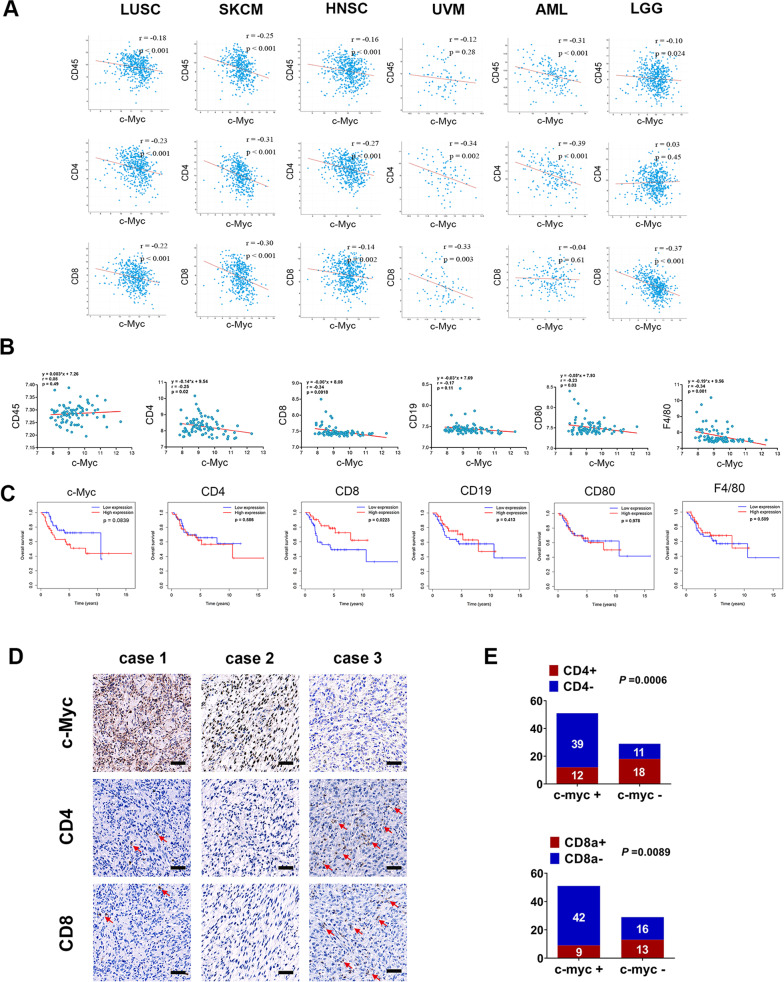
The expression of c-Myc is negatively correlated with T-cell infiltration in many solid tumors. **A** Analysis of the correlation between c-Myc expression and CD45, CD4, CD8 expression in LUSC, SKCM, HNSC, UVM, AML, and LGG (TCGA dataset). **B** Correlation between c-Myc expression and immune cell marker expression in osteosarcoma (GEO dataset, GSE33382). **C** The relationship between the expression of c-Myc, CD4, CD8, CD19, CD80, F4/80, and the prognosis of osteosarcoma (TARGET database). **D** Representative pictures of immunohistochemical staining of c-Myc, CD4, and CD8 in 80 human osteosarcoma samples. scale bar: 100 μm. Statistical comparisons were performed using Fisher’s exact test (**E**).

Previous studies reported that endogenous Myc maintains the tumor microenvironment and is associated with immune suppression [14, 15, 23], but little is known about the relationship between Myc overexpression and T cell infiltration within solid tumors. To investigate this, we performed a pan-cancer transcriptome analysis of the TCGA dataset. The results showed that the expression level of c-Myc was negatively correlated with those of CD45 (the pan-leukocyte marker), CD4 (T-helper cell marker), and CD8 (CTL marker) in several kinds of human malignancies, including lung squamous cell carcinoma, skin cutaneous melanoma, uveal melanoma, head and neck squamous cell carcinoma, and brain lower-grade glioma (Fig. 1A). Because osteosarcoma is a rare cancer type that is not part of TCGA, we reanalyzed a publicly available dataset involving 84 pretreatment high-grade osteosarcoma samples (GEO dataset GSE33382). We found that the c-Myc level showed a significant negative correlation with the CD4 and CD8 levels. However, there were no negative correlations between the expression levels of c-Myc and CD45 in the osteosarcoma samples analyzed (Fig. 1B). These results suggest that c-Myc may be involved in regulating the abundances of CD4+ and CD8+ T cell subsets rather than that of total lymphocytes in osteosarcoma. Next, we performed IHC staining to examine the expression of c-Myc, CD4, and CD8 in a cohort of 80 human osteosarcoma specimens. The results showed that c-Myc was highly expressed in more than half of the osteosarcoma samples (63.75%, 51/80) and exhibited a predominantly nuclear staining pattern (Fig. 1D, E). In contrast, positive staining for CD4 and CD8 was detected only in a small proportion of osteosarcoma tissues (37.5% and 27.5%, respectively), supporting the notion that osteosarcoma tumors are mainly immunologically “cold” [11, 24]. Moreover, CD4+ and CD8+ T cells were largely absent in the c-Myc-positive samples (Fig. 1E), which is consistent with our previous findings that there were inverse correlations between the expression level of c-Myc and those of CD4 and CD8. Then, we obtained transcriptome and clinical data of osteosarcoma patients from the TARGET database. Kaplan–Meier survival analysis revealed that high expression of c-Myc was associated with shorter survival in osteosarcoma, while patients bearing tumors with higher CD8 levels had significantly improved overall survival (*p* = 0.0223) compared with those bearing tumors with lower CD8 levels (Fig. 1C). Collectively, these results suggested that the expression of the c-Myc oncogene is negatively correlated with the abundances of CD4+ and CD8+ tumor-infiltrating lymphocytes (TILs) in human osteosarcoma.

### c-Myc inhibition reprogrammed the tumor immune microenvironment and showed marked antitumor efficacy against osteosarcoma

**Fig. 2:**
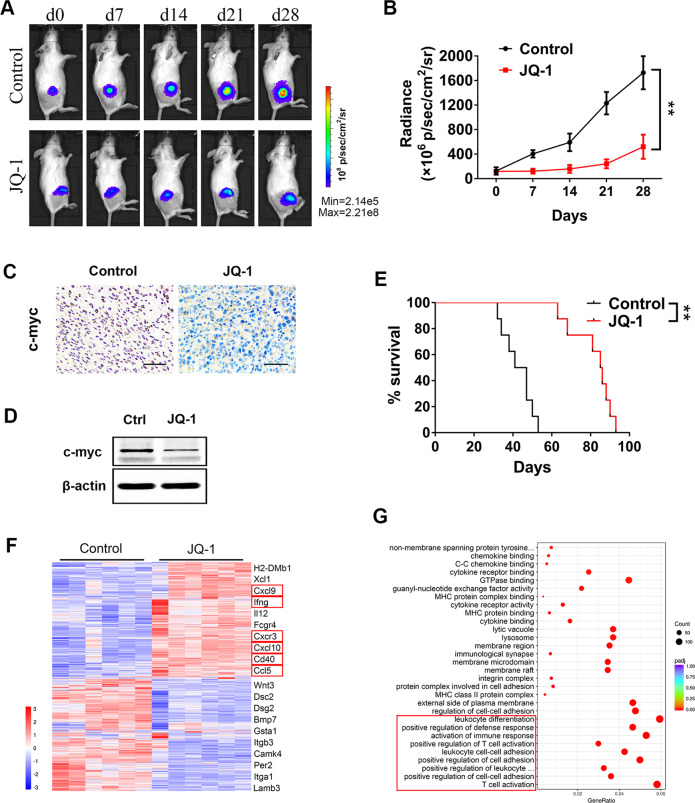
JQ-1 represses tumor growth and alters the tumor immune microenvironment in the K7M2 model. **A** Bioluminescence of K7M2 tumor after confirmation of tumor formation, tumor progression was monitored weekly. **B** Tumor burden was evaluated by total flux, *n* = 6. **C** Representative immunohistochemical staining of c-myc in K7M2 tumors, scale bar: 100 μm. **D** Western blot analysis of the levels of c-myc in K7M2 tumors, treated or untreated with JQ-1. **E** Kaplan–Meier curves showing the survival time of mice in (**A**). **F** Heat map showing the gene expression profile of differentially expressed genes based on their hierarchical clustering. Cd40 and several T cell-recruiting chemokines significantly upregulated in JQ-1 treated group are highlighted. **G** Significantly different terms in GO analysis.

To evaluate the immunomodulatory and antitumor effects of c-Myc inhibition in vivo, we established an immunocompetent syngeneic murine model of osteosarcoma using luciferase-expressing K7M2 cells [25]. Two weeks after K7M2 cell inoculation, tumor-bearing mice were treated with 50 mg/kg JQ-1, a BET bromodomain inhibitor specifically targeting c-Myc [26], once daily for 28 days. Some mice in each group were sacrificed on the 7th and 28th days of treatment, and the c-Myc protein level and transcriptome changes in K7M2 tumors were detected. The remaining mice were kept throughout the survival period, and tumor growth was assessed weekly by bioluminescence imaging during treatment (Fig. 2A). The results showed that JQ-1 administration significantly decreased the c-Myc protein level (Fig. 2C, D) and retarded tumor growth in the K7M2 model (Fig. 2B). Moreover, pharmacological inhibition of c-Myc prolonged the survival time of tumor-bearing mice (Fig. 2E), thus showing marked antitumor efficacy against osteosarcoma. Then, we performed Gene Ontology (GO) analysis on RNA-seq data of K7M2 tumors. Surprisingly, c-Myc inhibition mainly led to activation of multiple immune pathways, such as T cell activation, leukocyte cell–cell adhesion, and positive regulation of defense response, rather than to suppression of cell proliferation pathways (Fig. 2G). Among the genes most significantly upregulated by JQ-1 treatment, many have been reported as key regulators of the tumor microenvironment and immune response. For example, IFN-γ and IL-12 are immune activation markers, CCL5, CXCL9, and CXCL10 are T cell-recruiting chemokines, and CD40 is an immune costimulatory molecule (Fig. 2F). In summary, these data indicated that c-Myc inhibition probably exerts antitumor effects by reprogramming the tumor immune microenvironment of osteosarcoma, but the detailed mechanisms remain to be explored.

### c-Myc inhibition upregulated CD40/CD40L expression and induced T cell infiltration and activation in K7M2 tumors

**Fig. 3:**
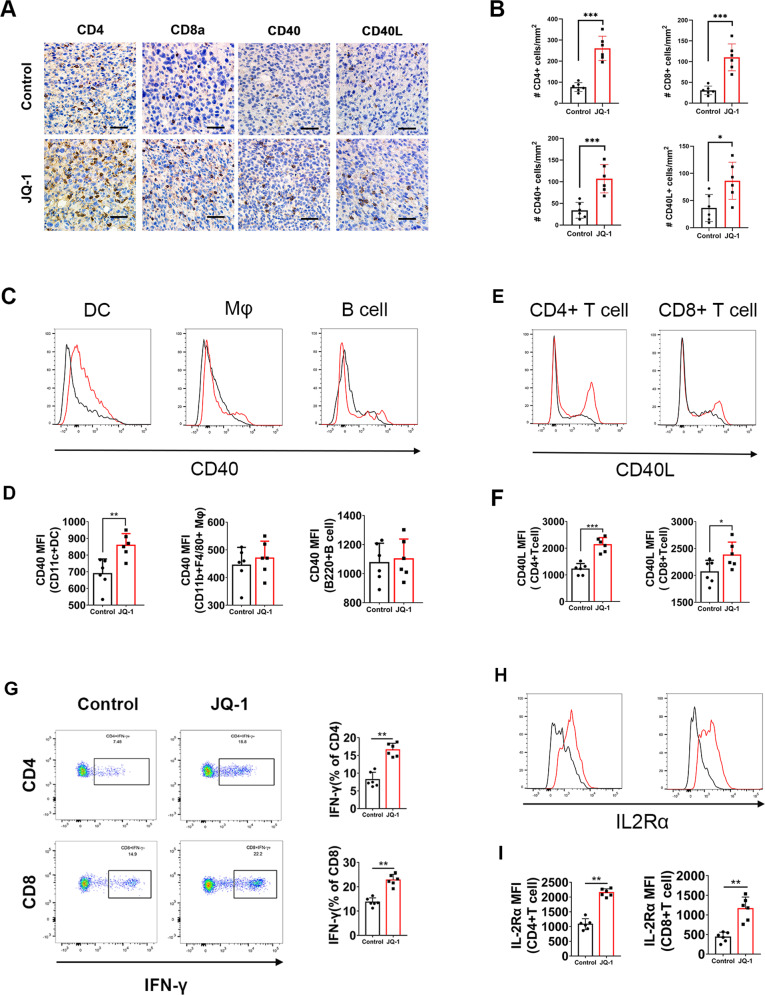
Treatment with JQ-1 increases T cell infiltration and upregulates CD40/CD40L expression in K7M2 tumor. **A** Representative immunohistochemical images of CD4, CD8a, CD40, CD40L in K7M2 tumors, treated with JQ-1 or DMSO (400×, scale bar: 100 μm). **B** Positive signals were counted in four randomly selected fields (400×) in each tumor section using ImageJ, *n* = 6. **C**, **D** Representative histogram showing CD40 expression on dendritic cells (CD45 + CD11c + MHC-II+), macrophages (CD45 + CD11b + F4/80+) and B cells (CD45 + CD3-CD11c−B220+). **E**, **F** Histogram overlay showing CD40 ligand expression on CD4+ T cells and CD8+ T cells. **G** Representative Pseudo-color plots of IFN-γ expression in CD4+ T cells and CD8+ T cell cells. **H**, **I** Representative histogram overlay showing IL2Rα expression on CD4+ T cells or CD8+ T cells.

Next, we performed IHC to examine the expression of CD4 and CD8 in K7M2 tumors. The results showed that the abundances of CD4+ and CD8+ cells inside tumors were dramatically increased after JQ-1 administration (Fig. 3A, B), indicating that pharmacological inhibition of c-Myc can enhance T cell infiltration in osteosarcoma. We also observed significant increases in the numbers of CD40+ and CD40L+ cells in the K7M2 tumor microenvironment (Fig. 3A, B), which confirmed the results of the RNA-seq analysis. Notably, previous reports demonstrated that CD40 is expressed on the surface of all APCs, including DCs, macrophages, and B cells, and that CD40L is preferentially expressed on T cells [27]. To investigate the cell type-specific expression patterns of CD40 and CD40L, we then performed flow cytometric analysis of K7M2 tumors to examine the levels of CD40 and CD40L on different immune cell types. The results showed that JQ-1 administration induced upregulation of CD40 on DCs rather than on macrophages and B cells (Fig. 3C, D) and increased the expression of CD40L on CD4+ and CD8+ T cells (Fig. 3E, F). Furthermore, we observed marked increases in the percentages of IFN-γ-producing CD4+ and CD8+ T cells in K7M2 tumors (Fig. 3G), suggesting that tumor-infiltrating T lymphocytes were activated upon c-Myc inhibition. IL-2Rα, which is a marker of the activated CD40/CD40L system [28], was also upregulated in CD4+ and CD8+ TILs (Fig. 3H, I). Taken together, these data indicated that c-Myc inhibition can upregulate CD40/CD40L expression and induce T cell infiltration and activation in osteosarcoma.

### CD40-CD40L interactions are essential for the therapeutic effects of c-Myc inhibitor

**Fig. 4:**
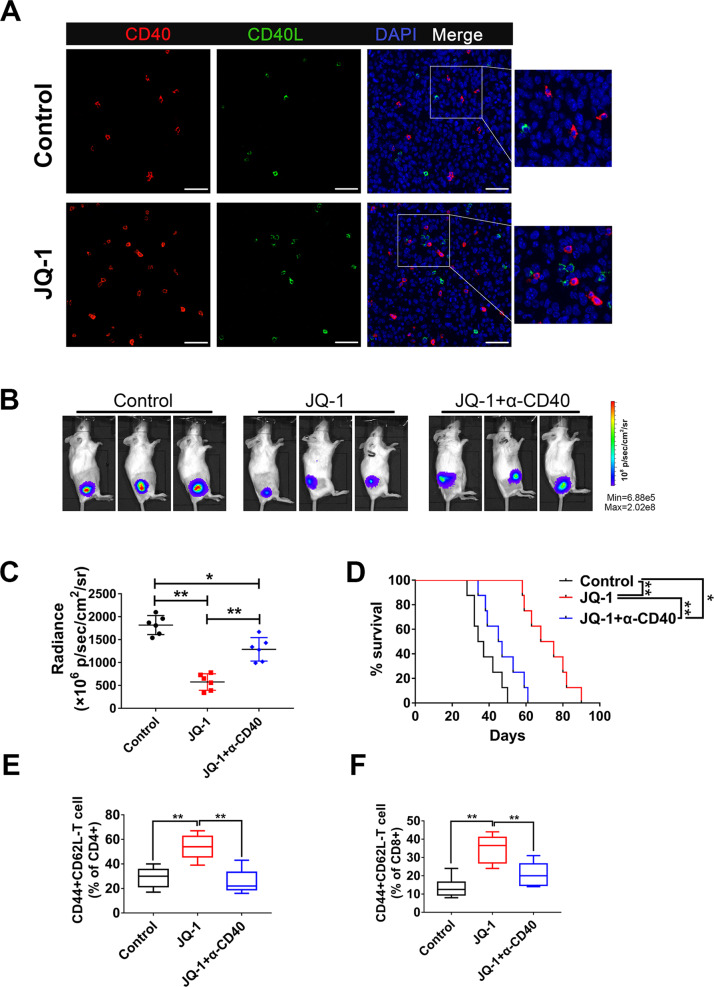
The therapeutic effects of JQ-1 depend on CD40-CD40L interaction. **A** Representative immunofluorescence staining of CD40 and CD40L in K7M2 tumors (400×, scale bar: 100 μm). **B**, **C** Representative bioluminescence of K7M2 model at day 28 post first treatment, tumor burdens were evaluated by total flux. **D** The long-term survival of mice treated with DMSO, JQ-1, or JQ-1 plus CD40L block antibody is shown. **E**, **F** The proportion of CD44 + CD62L-populations in CD4+ T cells or CD8+ T cells.

Previous studies have demonstrated that ligation of CD40 on the surface of APCs greatly enhances their costimulatory capacity and that CD40-CD40L interactions are vital in the delivery of T cell help for CTL priming [27]. Therefore, we asked whether CD40-CD40L interactions would contribute to JQ-1-induced T cell activation in osteosarcoma. To address this, double immunofluorescent staining was performed to detect the spatial distribution patterns of CD40 and CD40L within K7M2 tumors. The results showed that sporadic red (CD40) and green (CD40L) signals were scattered in control tumors, with a salient feature that CD40 and CD40L were not adjacent to each other (Fig. 4A, upper panel). In the JQ-1 treatment group, the positive signals of CD40 and CD40L were not only increased but also showed mutual proximity and interactions at many locations inside the tumors (Fig. 4A, lower panel), suggesting that c-Myc inhibition can enhance the CD40-CD40L interaction in the osteosarcoma microenvironment. To further study the functional role of CD40-CD40L, we used a blocking antibody to CD40 to disrupt the CD40-CD40L interactions in JQ-1-treated tumor-bearing mice. The results indicated that blockade of CD40/CD40L interactions markedly reduced the therapeutic effects of JQ-1 in the K7M2 model (Fig. 4B, C, D). Moreover, we observed a marked increase in the percentage of effector memory (CD44 + CD62L-) T cells in K7M2 tumors treated with JQ-1, and this effect was largely eliminated by CD40 blockade (Fig. 4E, F). Together, these results suggested that CD40-CD40L interactions are essential for JQ-1-induced T cell activation and the related therapeutic effects.

### c-Myc inhibition promoted tumor regression by increasing the expression and secretion of T cell-recruiting chemokines in osteosarcoma

**Fig. 5:**
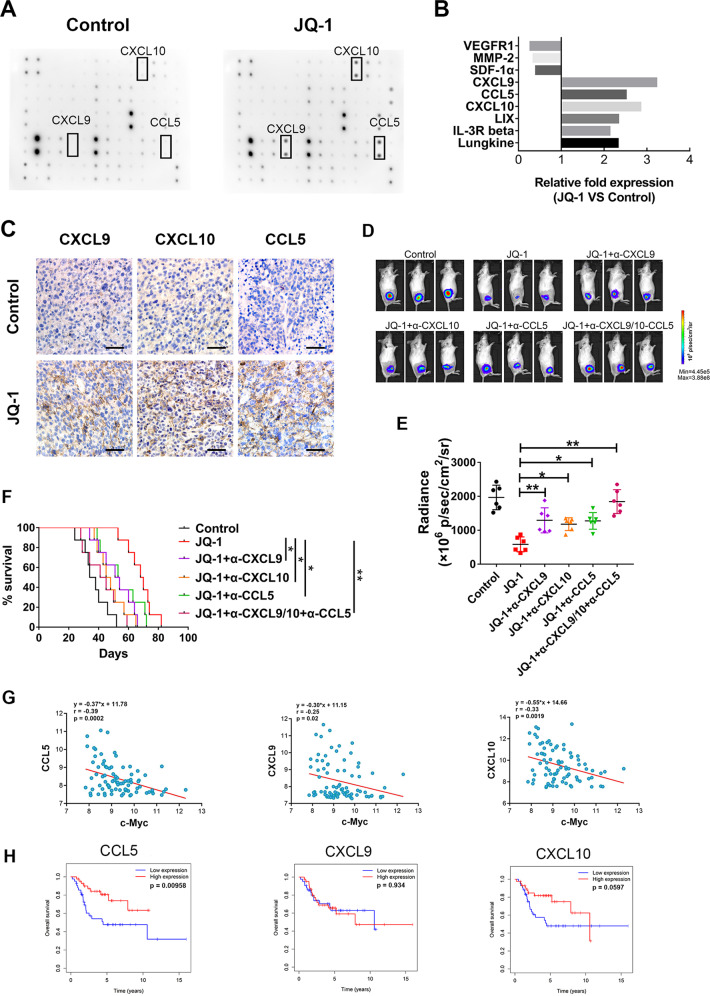
c-Myc inhibition promotes the secretion of CXCL9, CXCL10, and CCL5 in osteosarcoma. **A** Mouse cytokine array C-1000 was used to detect chemokine variations in the supernatants of K7M2 cells treated with DMSO or JQ-1, *n* = 3. **B** Statistical results of grayscale analysis, chemokines with a fold change >2 and *P* < 0.05 are shown. **C** Representative immunohistochemical images of CXCL9, CXCL10, and CCL5 in K7M2 tumors, treated with JQ-1 or DMSO (400×, scale bar: 100 μm). **D**, **E** Representative bioluminescence pictures of mice on day 28 after injection of chemokine neutralizing antibody, tumor burdens were evaluated by total flux, *n* = 6. **F** The long-term survival of mice treated with CXCL9, CXCL10, CCL5 neutralizing antibody. **G** Correlation analysis of c-Myc expression and CXCL9, CXCL10, CCL5 expression in human samples (GEO dataset). **H** The relationship between the expression of CXCL9, CXCL10, and CCL5 and the prognosis of osteosarcoma (TARGET database).

To investigate the mechanisms of JQ-1-induced T cell infiltration, we treated K7M2 cells with JQ-1 or DMSO in vitro and collected the supernatants. Then, cytokine secretion profiles of K7M2 cells were analyzed using the RayBio Mouse Cytokine Antibody Array. The results showed that JQ-1 treatment significantly increased the secretion of several T cell-recruiting chemokines, such as CCL5, CXCL9, and CXCL10 (Fig. 5A, B), which was in line with the results of the RNA-seq analysis (Fig. 2F). Furthermore, we performed IHC staining and found that the expression of CCL5, CXCL9, and CXCL10 was not only markedly enhanced but also widely distributed in K7M2 tumors treated with JQ-1 (Fig. 5C). To study the functional roles of these T cell-recruiting chemokines, we used neutralizing antibodies to antagonize the expression of CCL5, CXCL9, and CXCL10 in JQ-1-treated tumor-bearing mice. The results indicated that in vivo neutralization of CCL5, CXCL9, or CXCL10 significantly reduced the therapeutic effects of JQ-1 in the K7M2 model and that simultaneous neutralization of all three chemokines showed an obvious synergistic effect (Fig. 5D, E, F).

Given that the expression of CCL5, CXCL9, and CXCL10 could be upregulated by a c-Myc inhibitor in the K7M2 model, we then asked whether this regulatory relationship also existed in human osteosarcoma. To answer this question, we reanalyzed the abovementioned GEO dataset and TARGET database. The results showed that there were significant negative correlations between the c-Myc expression level and those of CCL5, CXCL9, and CXCL10 in human osteosarcoma samples. Kaplan–Meier survival analysis revealed that patients bearing tumors with higher CXCL9 levels did not show a difference in overall survival compared with those bearing tumors with lower CXCL9 levels, suggesting that CXCL9 is not a good independent prognostic factor in osteosarcoma. However, we found that high expression levels of CCL5 and CXCL10 were associated with improved survival in patients with osteosarcoma, although the association for CXCL10 was not considered statistically significant (*p* = 0.0597). Collectively, these results suggested that c-Myc inhibition can promote the regression of osteosarcoma by increasing the expression and secretion of CCL5, CXCL9, and CXCL10 in tumor tissues.

### Combination treatment with a c-Myc inhibitor and an anti-PD-1 antibody exhibited a synergistic therapeutic effect in osteosarcoma that was dependent on CD4+ and CD8+ T cells

**Fig. 6:**
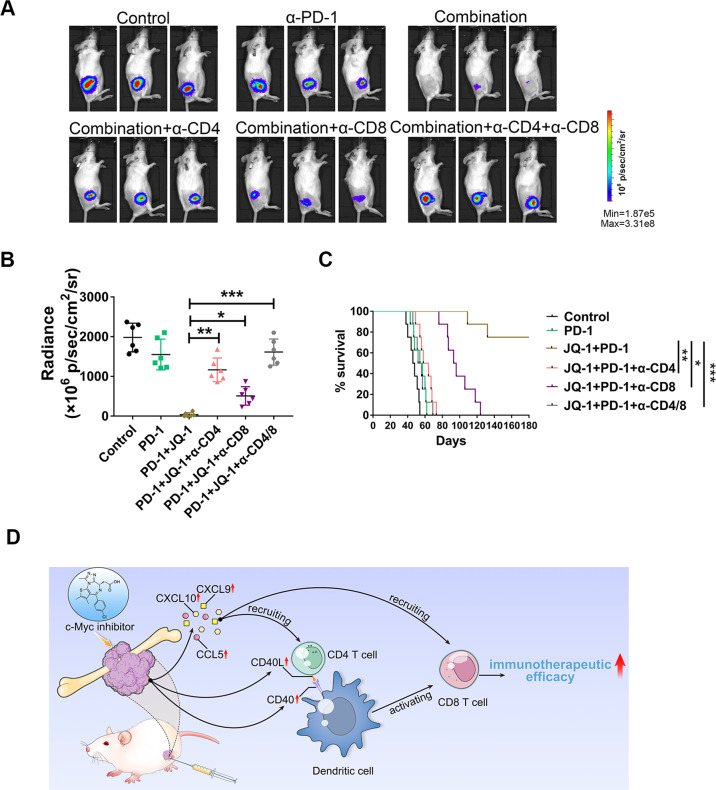
Either CD4+ T cell or CD8+ T cell depletion impaired the antitumor effects of combination therapy. **A**, **B** Representative bioluminescence images of K7M2 tumors at day 28 post treatment initiation (*n* = 6), tumor burdens were evaluated by total flux. **C** Kaplan–Meier curves showing survival of tumor-bearing mice treated with DMSO, anti-PD-1 antibody, combination therapy, and combination therapy plus T cell depletion. **D** Schematic model showing how c-Myc inhibitor reprograms tumor immune microenvironment in osteosarcoma. On the one hand, JQ-1 can promote T cell trafficking into tumors by increasing the expression and secretion of T cell-recruiting chemokines. On the other hand, JQ-1 is capable of facilitating crosstalk between antigen-presenting dendritic cells and T cells through the CD40/CD40L costimulatory pathway, leading to activation of tumor-specific CTLs.

Based on the above findings that c-Myc inhibition can enhance T cell infiltration and activate the CD40/CD40L costimulatory pathway, we speculated that the combination of a c-Myc inhibitor and an anti-PD-1 antibody might have a synergistic therapeutic effect in osteosarcoma. To test this hypothesis, mice bearing K7M2 tumors were injected intraperitoneally with an anti-PD-1 mAb (200 μg in 100 μL per injection), JQ-1 (50 mg/kg in 200 μL) plus the anti-PD-1 mAb, or saline (200 μL per injection). JQ-1 was administered once daily, and the anti-PD-1 mAb was administered every 3 days for a total of 21 days. In animals treated with the anti-PD-1 mAb alone, tumor growth was not significantly suppressed compared with that in saline-treated mice, as determined by bioluminescence signals (Fig. 6A, B). However, combined treatment with the anti-PD-1 mAb and JQ-1 resulted in more pronounced tumor regression and ultimate cure in three-quarters (6/8) of the mice, exhibiting much better antitumor efficacy against osteosarcoma than either monotherapy alone (Fig. 6A, B, C). Given that c-Myc inhibition exerted antitumor effects by enhancing T cell infiltration and activation, we then asked whether CD4+ and CD8+ T cell subsets play pivotal roles in the combination treatment of osteosarcoma. To address this question, T cell subsets were depleted in tumor-bearing mice using depleting anti-CD4 and anti-CD8 mAbs. The results showed that in vivo depletion of CD4+ or CD8+ T lymphocytes severely impaired the antitumor efficacy of combination therapy with JQ-1 and anti-PD-1 mAb. Notably, simultaneous depletion of CD4+ and CD8+ T cells almost completely abolished the antitumor efficacy of the combination therapy, with an efficacy level comparable to that in the control group (Fig. 6A, B, C). In summary, these results indicated that combination treatment with a c-Myc inhibitor and an anti-PD-1 antibody exhibited a synergistic therapeutic effect in osteosarcoma that was dependent on CD4+ and CD8+ T cells.

## Discussion

The treatment of primary osteosarcoma with wide resection yielded favorable outcomes. However, improved treatment strategies are needed for pelvic and advanced cases [29]. It has been more than 40 years since the discovery of the c-Myc proto-oncogene. The protein encoded by the c-Myc gene mainly functions as a transcription factor that regulates the expression of thousands of downstream genes primarily involved in proliferation, cell survival, self-renewal, metabolism, invasiveness, and angiogenesis. As a master regulator of many important biological pathways, the c-Myc oncogene contributes to the genesis of multiple malignancies [13, 30]. It is estimated that c-Myc expression is elevated or deregulated in up to 70% of human cancers, which makes c-Myc a valuable therapeutic target. Preclinical studies have demonstrated that aberrant activation of c-Myc can elicit many of the hallmarks of cancer, while c-Myc inactivation results in sustained tumor regression, a phenomenon that could be attributed to oncogene addiction [13]. Intriguingly, c-Myc was recently found to be able to drive tumor growth not only through its intrinsic effects on cellular proliferation but also through its regulation of the tumor microenvironment and immune evasion, indicating that c-Myc also functions as a key regulator of the immune response [31]. In a mouse model of pancreatic islet tumors, short-term systemic c-Myc inhibition was sufficient to trigger collapse of the tumor microenvironment, with concomitant tumor vasculature collapse and reduced infiltration of immunosuppressive macrophages [15]. Furthermore, c-Myc has been found to upregulate the expression of immune checkpoint molecules, such as CD47 and PD-L1, thus leading to dysfunction of macrophages and T cells [14]. c-Myc also cooperates with Ras to regulate the secretion of CCL9 and IL-23, thereby promoting the recruitment of immunosuppressive macrophages and the exclusion of adaptive T and B cells, which in turn facilitates tumor immune escape [23]. In addition, recent reports showed that c-Myc amplification is associated with low immunogenicity of cancer cells and that targeting c-Myc can markedly induce tumor immunogenicity [32]. The abovementioned results suggested that c-Myc plays a versatile role in tumor immune suppression. However, in the field of osteosarcoma, most studies have focused on the cell-autonomous effects of c-Myc on cell growth, apoptosis, cancer metabolism, etc., while the relationship between tumor immune suppression and c-Myc overexpression in osteosarcoma has remained largely unexplored. In this study, we found that c-Myc was highly expressed in a significant proportion of osteosarcoma samples tested (Fig. 1D, E), which is in line with previous reports [21, 22]. Unexpectedly, RNA-seq analysis revealed that pharmacological inhibition of c-Myc in osteosarcoma mainly resulted in activation of multiple immune pathways, including T-cell activation, leukocyte cell–cell adhesion, and positive regulation of defense response, rather than suppression of cell proliferation pathways (Fig. 2G). This phenomenon is probably due to the use of an immunocompetent syngeneic murine model of osteosarcoma in the present work, which enabled us to investigate and predict antitumor immune responses more faithfully and comprehensively [33].

In a recent study, Kazuhiko Hashimoto and colleagues surveyed and characterized the expression of CD4, CD8, PD-1, and PD-L1 in osteosarcoma cases in detail. They found that the PD-1/PD-L1 immune checkpoint system, involving CD4 and CD8, plays an important role in the pathogenesis of osteosarcoma [34]. In line with this, our work revealed that depletion of either CD4+ T cells or CD8+ T cells abrogated the therapeutic effects of JQ-1 plus PD-1 antibody (Fig. 6A). Furthermore, our work demonstrated that pharmacological inhibition of c-Myc in osteosarcoma can promote T cell trafficking into tumor beds by increasing the expression and secretion of CCL5, CXCL9, and CXCL10 (Fig. 5). To our knowledge, all three chemokines have been reported to play key roles in immune cell chemotaxis and immunomodulation, usually in a synergistic manner. For instance, coexpression of CCL5 and CXCL9 shapes immunoreactive and immunoresponsive tumors with increased CTL infiltration, and an in vivo study demonstrated that forced CCL5 expression can sustain T cell infiltration and CXCL9 expression in solid tumors [35]. Moreover, chemotherapy induces intratumoral expression of CCL5, CXCL9, and CXCL10 in cutaneous melanoma, favoring T cell infiltration and tumor control [36]. Transcriptional profiling of human colorectal cancer (CRC) tissues revealed that active secretion of CCL5 and CXCL10 from the tumor microenvironment is closely associated with GZMB + CD8+ T cell infiltration, suggesting a critical role of CCL5 and CXCL10 in CTL recruitment [37]. In another study, CCL5, CXCL9, and CXCL10 were defined as chemokine gene signatures for CRC infiltration by CTLs and T-helper (Th)1 cells [38], consistent with the findings from our current study. However, the regulatory mechanisms underlying the expression of T cell-recruiting chemokines remain largely unknown. In the present work, we first identified the relationship between c-Myc inhibition and upregulation of CCL5, CXCL9, and CXCL10 in osteosarcoma. We also found that c-Myc inhibition can facilitate crosstalk between antigen-presenting DCs and T cells through the CD40/CD40L costimulatory pathway. Finally, we concluded that c-Myc inhibition is capable of promoting T cell infiltration and activation in osteosarcoma in multiple ways, thus delivering a one-two punch for modulating the tumor immune microenvironment (Fig. 6D).

## Materials and methods

### Bioinformatics analysis based on public database

RNA-seq data for pan-cancer analysis were downloaded from The Cancer Genome Atlas (TCGA, http://cancergenome.nih.gov/). Gene expression data for osteosarcoma were downloaded from the Therapeutically Applicable Research To Generate Effective Treatments database (TARGET, https://ocg.cancer.gov/programs/target) and the Gene Expression Omnibus (GEO) database. Correlation analysis was carried out using Pearson correlation analysis. The clinical data related to overall survival were downloaded from the TARGET database, and Kaplan–Meier analysis was used to analyze the relationship between gene expression levels and the prognosis of patients. R4.0.2 was used for statistical analysis, and differences were considered to be statistically significant when *p* < 0.05.

### Mouse and tumor model

Six-week-old female BALB/c mice were purchased from the Model Animal Research Center of Nanjing University (Nanjing, China). The K7M2 mouse osteosarcoma cell line was obtained from the Type Culture Collection of the Chinese Academy of Science.

To establish the in vivo tumor model, 5 × 10^6^ luciferase-tagged K7M2 cells were subcutaneously injected into the flanks of BALB/c mice. Tumor burdens were monitored every week by an in vivo imaging system. All animal experiments were approved by the Institutional Animal Care and Use Committee of Xi’an Jiaotong University.

### Inhibitor and therapeutic antibody

JQ-1, a BET bromodomain inhibitor specifically targeting c-Myc (MCE, HY-13030), was intraperitoneally administered daily (50 mg/kg) 20 days after tumor inoculation. At the same time, an anti-PD-1 monoclonal antibody (BioXCell, RMP1-14, 200 µg per mouse) was intra-abdominally injected every 3 days.

### Immunohistochemistry (IHC) and immunofluorescence (IF)

IHC and IF were performed on paraffin-embedded samples. For human samples, the primary antibodies used included rabbit anti-human c-myc (Abcam, ab32072), rabbit anti-human CD4 (CST, 48274), and rabbit anti-human CD8a (CST, 85336). For mouse tissues, the primary antibodies used included rabbit anti-mouse c-myc (Abcam, ab32072), rabbit anti-mouse CD8a (CST, 98941), and rabbit anti-mouse CD4 (CST, 25229). ImageJ software was used for image analysis. For IF staining, paraffin-embedded sections were stained with rabbit anti-mouse CD40 (Abcam, ab273098) and hamster anti-mouse CD40L (Abcam, ab99894) antibodies. DAPI was used for nucleic acid staining.

### Western blotting

K7M2 tumors were lysed, and proteins were separated by SDS–PAGE and transferred onto polyvinylidene membranes. After blocking in 5% nonfat milk, membranes were incubated with an anti-mouse c-myc antibody (Abcam, ab32072), and an anti-mouse β-Actin antibody (Abcam, ab8227) was used to detect β-Actin as the loading control. HRP-conjugated goat anti-rabbit IgG (Abcam, ab97051) was used as the secondary antibody. Finally, images were acquired using an ECL system (Amersham Pharmacia Biotech).

### RNA sequencing and bioinformatics analysis

Mice were sacrificed 7 days after treatment initiation. Tumor tissues were quickly obtained and stored in RNAlater solution after the mice were sacrificed. Following RNA extraction (TRIzol™ Plus RNA Purification Kit, Invitrogen), a NEBNext® Ultra™ Directional RNA Library Prep Kit for Illumina® (BioLabs) was used for cDNA synthesis. RNA sequencing was then performed on the HiSeq X ten PE150 NovaSeq 6000 platform.

After quality control, the data acquired from RNA sequencing were used for bioinformatics analysis. DESeq2 was used to identify differentially expressed genes between the two groups. Cluster Profiler software was used for Gene Ontology (GO) analysis, which included biological process, cellular component, and molecular function ontologies. For GO functional enrichment analysis, a padj value of <0.05 is considered the threshold for significant enrichment.

### Flow cytometry

Tumor-infiltrating lymphocyte isolation and other procedures, including stimulation, fixation, permeabilization, and intracellular staining, were described previously [39]. Antibodies used for surface marker staining included anti-CD45 (BioLegend, clone: 30-F11), anti-CD3 (BioLegend, clone: 17A2), anti-CD11c (BioLegend, clone: N418), anti-MHC-II (Invitrogen, clone: M5/114.15.2), anti-CD11b (BioLegend, clone: M1/70), anti-F4/80 (BioLegend, clone: BM8), anti-B220 (BioLegend, clone: RA3-6B2), anti-CD4 (BD Horizon, clone: RM4-5), anti-CD8 (BD Pharmingen, clone: 53-6.7), anti-CD40 (BioLegend, clone: 3/23), anti-CD40L (BioLegend, clone: SA047C3), anti-IL2Rα (BioLegend, clone: 3C7), and anti-CD44 (BioLegend, clone: IM7), and anti-CD62L (BioLegend, clone: MEL-14) antibodies. For cytokine staining, an anti-IFN-γ antibody (BD Pharmingen, clone: XMG1.2) was used after TIL fixation and permeabilization. Data were acquired on a BD FACSCanto II flow cytometer (BD Biosciences) and analyzed with FlowJo software (Tree Star).

### Mouse cytokine and chemokine array analysis

K7M2 cells were plated in six-well plates at 1 × 10^6^ cells per well and cultured for 24 h in complete growth medium. Then, the cells were washed with PBS and cultured in serum-free DMEM plus 250 nmol/L JQ-1 or DMSO. Twenty-four hours later, the supernatants were collected. Mouse cytokine and chemokine arrays (Ray Biotech, AAM-CYT-1000) were used for protein detection. Finally, the arrays were scanned in an LAS-500 (Fuji) imager. Relative protein levels were assessed by grayscale analysis.

### In vivo neutralization, blockade, and cell depletion

To neutralize CCL5, CXCL9, and CXCL10 in vivo, anti-mouse CCL5 (R&D Systems, MAB478, 50 μg per mouse), anti-mouse CXCL9 (R&D Systems, AF-492-NA, 50 μg per mouse), anti-mouse CXCL10 (R&D Systems, MAB466, 50 μg per mouse) or a mixture of these antibodies were intraperitoneally injected twice weekly.

For CD40/CD40L signal blockade in vivo, an anti-mouse CD40L monoclonal antibody (BioXCell, MR-1, 300 μg per mouse) was intraperitoneally administered twice weekly.

To deplete CD4+ T cells or CD8+ T cells, mice were injected intraperitoneally with 400 μg of an anti-mouse CD4 antibody (BioXCell, GK1.5) or an anti-mouse CD8 antibody (BioXCell, YST-169.4) every 3 days.

### Statistical analysis

GraphPad Prism 8 was used for statistical analysis, and all data are presented as the mean ± SD. Differences between two groups were analyzed by unpaired *t* test, and one-way ANOVA was used for comparisons among three or more groups. The Kaplan–Meier method was used to plot survival curves, and the statistical significance of differences in overall survival between groups was determined by the log-rank test. Significance was established as follows: *p < 0.05; **p < 0.01; ***p < 0.001.

## Data Availability

The datasets used and analyzed during the current study are available from the corresponding authors on reasonable request.
